# Clinical assessment of blood glucose responses to 300-mg dose of L-theanine, an amino acid unique to green tea, in a fixed-sequence, two-period trial

**DOI:** 10.1186/s40780-026-00562-6

**Published:** 2026-03-18

**Authors:** Shinnosuke Yamaura, Koichi Kawada, Kyosuke Uno, Reiko Konishi, Akira Mukai, Satoshi Tada, Hitoshi Okada, Takashi Majima, Koji Komori, Nobuyuki Kuramoto, Kou Kawada

**Affiliations:** 1 Laboratory of Clinical Pharmacology and Therapeutics, Faculty of Pharmaceutical Sciences, Setsunan University, 45-1 Nagaotoge-cho, Hirakata, 573-0101 Osaka Japan; 2 Laboratory of Molecular Pharmacology, Faculty of Pharmaceutical Sciences, Setsunan University, 45-1 Nagaotoge-cho, Hirakata, 573- 0101 Osaka Japan; 3 Department of Medical Technology, Kagawa Prefectural University of Health Sciences, 281-1 Mure-cho, Takamatsu, 761-0123 Kagawa Japan; 4 Faculty of Sports and Health Science, Department of Nursing, Daito Bunka University, 560 Iwadono, Higshimatuyama, 355-8501 Saitama Japan

**Keywords:** L-theanine, Diabetes, Glucose tolerance test, Blood glucose level, Urinary glucose

## Abstract

**Background:**

L-theanine (N-ethyl-γ-glutamine), an amino acid found in tea leaves and known for contributing to their umami taste, is widely marketed as a supplement for promoting relaxation. Recently, potential effects on blood glucose regulation have been reported. This study evaluated the effect of oral L-theanine intake on blood glucose levels using an oral glucose tolerance test (OGTT) in healthy adults.

**Methods:**

Healthy adult participants ingested 300 mg of L-theanine 15 min before the OGTT. Blood glucose levels were measured at 0 (baseline), 30, 60, 90 and 120 min after the start of the OGTT. A fixed-sequence, two-period trial was conducted: at Visit 1 (pre-intervention), the OGTT was performed without L-theanine, whereas at Visit 2 (intervention), the participants ingested 300 mg of L-theanine before undergoing the OGTT. In addition to blood samples, urine samples were collected whenever the participants felt the need to void and analyzed for glucose, sodium ions, and amino acids.

**Results:**

Thirty-nine healthy adults participated in the study. Although blood glucose levels tended to be lower at Visit 2 than at Visit 1, there was no statistically significant difference in the primary endpoint, defined as the difference between the peak glucose level and baseline. Thirty minutes after the start of the OGTT, corresponding to the time when serum L-theanine reached its peak, urinary glucose excretion tended to be higher and urinary sodium ions tended to be lower at Visit 2, although the difference was not statistically significant. Urinary excretion of glutamic acid, aspartic acid, and several other amino acids significantly increased during Visit 2.

**Discussion:**

L-theanine may exert a glucose-lowering effect through previously reported mechanisms; however, the effect observed in this study was extremely small and unlikely to raise clinical concerns. Further research is required to clarify the potential effects of long-term L-theanine consumption.

## Introduction

L-Theanine, which is present in the leaves of *Camellia sinensis*, is a non-proteinogenic neutral amino acid with a structure similar to that of L-glutamine and is known as one of the umami components of tea [1, 2]. Popular teas, such as gyokuro and matcha, contain high levels of L-theanine, and in recent years, they have been marketed as supplements to promote relaxation and improve sleep quality. In the body, ingested L-theanine exists primarily in its free form and is metabolized to glutamate and ethylamine by glutaminase [3].

Studies using mice have reported that L-theanine suppresses stress-induced brain atrophy [4], normalizes aberrant neuroexcitation in the hippocampus [5] and increases the concentrations of acetylcholine, γ-aminobutyric acid, and serotonin in the brain [6], suggesting anxiolytic effects and improvements in sleep quality. Since 2020, clinical trials have also increased, and studies administering approximately 200 mg of L-theanine to healthy adults have measured electroencephalogram (EEG), salivary cortisol levels, sleep duration, and cognitive test scores, reporting its effectiveness against stress [7–10]. Notably, clinical trials have been conducted in patients with psychiatric disorders to explore its potential as an adjunct therapy. For example, Sarri et al. administered 450–900 mg of theanine in addition to regular medications to 46 patients with generalized anxiety disorder and reported improvements in sleep satisfaction [11]. Kahathuduwa et al. administered L-theanine (2.5 mg/kg) combined with caffeine to five boys aged 8–15 years with ADHD and reported improvements in attention and cognition [12]. Sakamoto et al. discussed the potential effectiveness of continuous L-theanine intake in schizophrenia [13].

In addition to its central nervous system-mediated antistress effects, L-theanine has also attracted attention for its potential anti-obesity and antidiabetic effects [14, 15]. In 2019, a cohort study by Ninomiya et al. reported that L-theanine significantly reduced the risk of type 2 diabetes in association with increased blood ethylamine levels [16]. Inspired by this report, we demonstrated that L-theanine promotes urinary glucose excretion in mice [17]. Our findings demonstrated that the glucose-lowering effect we observed may involve competitive inhibition of sodium-dependent neutral amino acid transporters in the renal proximal tubules. Increased substrate load on these transporters may lead to a localized reduction in sodium availability. Based on this mechanism, we proposed a hypothesis that the activity of sodium–glucose cotransporters (SGLTs), which also rely on sodium as a driving force, may be attenuated, thereby suppressing renal glucose reabsorption. However, while high-dose L-theanine (1000 mg/kg) sufficiently lowered blood glucose levels, the effect was weak at 100 mg/g.

Because SGLTs targeted by L-theanine also exist in humans, it is possible that the same mechanism lowers blood glucose levels. However, no studies evaluating the effects of L-theanine on blood glucose have been reported, and neither commercially available supplements nor ongoing clinical trials have adequately addressed its potential risks. Among reports evaluating adverse effects of L-theanine, those by Ozeki et al. and Kurihara et al. are important [18, 19]. These studies examined the safety of excessive intake by administering 840–1000 mg daily, divided into three or four doses, over 4 weeks. They reported no differences compared with a placebo in standard biochemical tests, such as AST, ALT, and albumin, or in gastrointestinal symptoms or other adverse events [18]. Although Ozeki et al. measured blood glucose and HbA1c levels and found no significant differences, the timing of sample collection and HbA1c values of male participants were not specified. Thus, no clinical trials have specifically focused on the glucose-lowering effects of L-theanine and discussions regarding its potential role in novel approaches to diabetes and its influence on diabetes treatment remain insufficient. Accordingly, in this study, we performed an oral glucose tolerance test (OGTT) in healthy adults to evaluate the effects of a commercially available supplement containing 300 mg of L-theanine on blood glucose levels, and to examine whether information on the habitual intake of green tea or L-theanine may represent a relevant factor influencing diabetes and its clinical management.

## Methods

### Reagents

The formulation administered to participants was a commercial product (“Cha-Theanine”) containing 300 mg of L-theanine as its sole active ingredient, purchased from Hoshino Kagaku Co., Ltd. (Kyoto, Japan). The carbohydrate solution required for the OGTT, “Trelan G75,” containing 75 g of glucose, was obtained from Yoshindo Inc. (Toyama, Japan). Reagents for the HPLC analysis of amino acids in blood and urine were purchased from FUJIFILM Wako Pure Chemical Corporation (Osaka, Japan). Blood glucose measurements of capillary blood obtained via finger prick were performed using a self-monitoring blood glucose (SMBG) meter ”Medisafe Fit Pro II” and sensor chips purchased from Terumo Corporation (Tokyo, Japan). The lancet device (“Safe-T-Pro Plus ”) used for finger puncture was purchased from Roche Diagnostics (Tokyo, Japan).

### Participants

Between January and July 2025, participants were recruited through the university’s web portal. The recruitment notice stated that eligible participants must be at least 18 years old; age, sex, height, and body weight were recorded, and blood and urine samples were collected. Individuals who met any of the following four criteria were excluded:

1. A history of or current treatment for abnormal renal or hepatic function.
2. A history of or current treatment for diabetes mellitus or related disorders.
3. Ongoing pharmacological treatment for any disease other than those listed in 1 and 2.
4. Current use of health foods or supplements presumed to affect blood glucose levels.

### Intervention

**Fig. 1:**
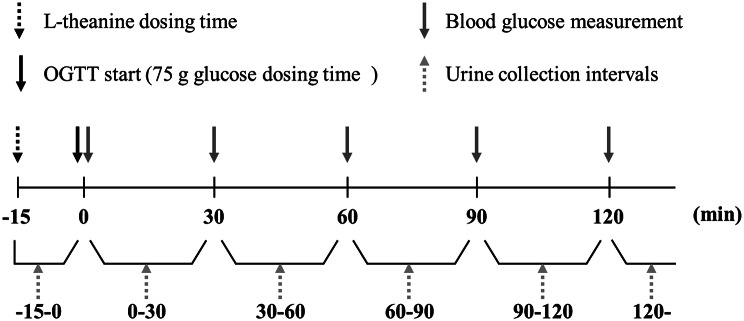
Timeline of the OGTT, blood glucose measurements, and urine collection

Participants were informed in writing about the study procedures, including fasting after 22:00 on the previous day, and provided written informed consent (Visit 0: informed consent). Each participant visited the laboratory twice within an 8–9 day period, and an OGTT was performed at each visit. The OGTT was conducted without L-theanine intake at Visit 1 (pre-intervention). During Visit 2 (intervention), the participants ingested a granule formulation containing 300 mg L-theanine, and the OGTT was initiated 15 min after ingestion. All study procedures were conducted after confirming that all participants were in a fasting state, in the morning during both visits. Participants consumed 75 g of glucose solution, and blood glucose levels were measured immediately after ingestion (0 min) and at 30, 60, 90, and 120 min thereafter, for a total of five measurements (Fig. 1). The blood glucose level at 0 min at each visit was defined as the baseline for that visit for each participant. Capillary blood was collected by the participants using a lancet device, and glucose levels were measured immediately. Blood glucose measurements were obtained by allowing a small amount of blood that emerged on the fingertip to be drawn into the SMBG device. Fingertip puncture and blood glucose measurement were performed by the participants themselves under the assistance and supervision of two trained staff members. Approximately 50 µL of additional blood was collected for amino acid analysis. In addition, to quantify urinary glucose, L-theanine, and other amino acids, participants collected urine samples as needed from immediately before the start of the OGTT through the approximately 2-hour period during which the OGTT was conducted.

The study design was approved by the Research Ethics Committee for Studies Involving Human Participants at Setsunan University in accordance with institutional guidelines (Approval No. 2024-69).

### Laboratory tests

Blood and urine samples were allowed to stand on ice for 1 h and then centrifuged at 3000 × *g* for 10 min (blood) or 30 min (urine). Supernatants were collected as analytical samples. Serum and urine supernatants were stored at − 80 °C until analysis. Urinary glucose, sodium ion, and creatinine levels were analyzed using the “TBA-120FR Sora Edition” (Canon Medical Systems Corporation, Tochigi, Japan). Sodium ions were measured using the ion-selective electrode method, glucose using the hexokinase–G6PDH method; and creatinine using a peroxidase-based enzymatic method involving creatinine amidinohydrolase. Serum and urinary L-theanine and other amino acids were quantified using the PITC-HPLC method as previously described [20].

### Statistical analyses

All data were compiled using Microsoft Excel 2019 (Microsoft Corp., Redmond, WA, USA). Statistical analyses were performed using the JMP Pro 18 software (SAS Institute Inc., Cary, NC, USA). Between-group variability in blood glucose levels at Visit 1 and Visit 2 was analyzed using paired t-test. Urinary parameters and amino acid levels were analyzed using the Wilcoxon signed-rank test to calculate p-values. A p-value < 0.05 was considered statistically significant. All measurements obtained from the 39 participants were included in the analysis, with no exclusion of outliers.

## Results

### Participants

Forty individuals were enrolled and provided written informed consent. One participant exhibited a urinary glucose concentration exceeding 300 mg/dL at Visit 1; this individual was advised to seek medical evaluation and was excluded from the study. The participant characteristics are summarized in Table 1. The participants consisted of 23 females and 16 males, aged 18–47 years, with a mean age of 23.9 ± 6.0 years and a median of 23 years. Height ranged from 1.51 to 1.80 m, with a mean of 1.64 ± 0.09 m and a median of 1.65 m. Body weight ranged from 39.9 to 83.0 kg, with a mean of 56.8 ± 10.7 kg and a median of 55 kg. BMI ranged from 16.1 to 29.4, with a mean of 21.0 ± 3.1 and a median of 20.7.

**Table 1:** Participant characteristics at Visit 1 (pre-intervention)

Population demographics (*n* = 39)	Mean ± SD Median (Min-Max)
Age (years)	23.9 ± 6.0 23 (18–47)
Female Male	23 (59.0%) 16 (41.0%)
Height (m)	1.64 ± 0.09 1.65 (1.51–1.80)
Weight (kg)	56.8 ± 10.7 55 (39.9–83.0)
Body Mass Index (kg/m²)	21.0 ± 3.1 20.7 (16.1–29.4)
Fasting glucose level (mg/dL)	102.9 ± 6.4 102 (92–119)

n, number of participants; SD, standard deviation; min, minimum; max, maximum

### Blood glucose levels in OGTT

**Fig. 2:**
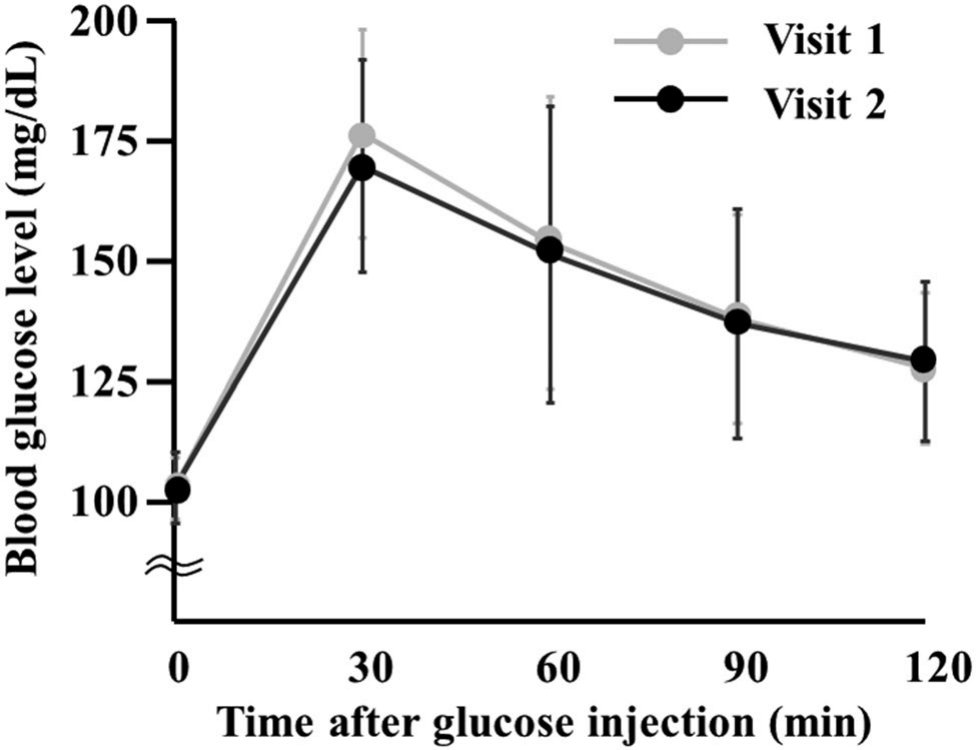
Time-course of Blood Glucose Concentrations During the OGTT. Values are presented as mean ± SD. The sample sizes were 39 for the control condition (visit 1) and 39 for the L-theanine condition (visit 2)

Blood glucose values at 0, 30, 60, 90, and 120 min during the OGTT in Visit 1 were 102.9 ± 6.4, 176.5 ± 21.6, 153.9 ± 30.5, 138.1 ± 21.7, and 127.7 ± 15.7 mg/dL, respectively. Corresponding values in Visit 2 were 102.9 ± 7.4, 169.8 ± 22.1, 151.5 ± 30.9, 137.2 ± 23.8, and 129.2 ± 16.6 mg/dL, showing no significant differences compared to those at Visit 1 (Fig. 2).

**Fig. 3:**
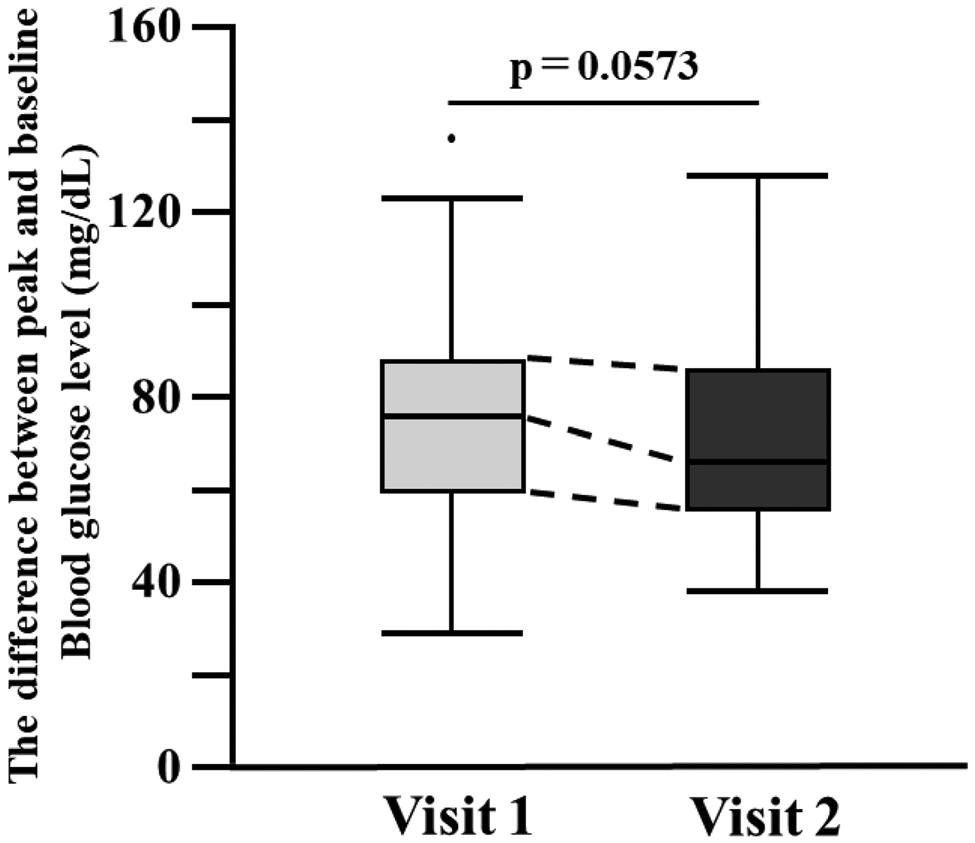
Comparison of peak-minus-baseline blood glucose values. For each participant, the increase in blood glucose levels was calculated by subtracting the baseline value from the peak OGTT glucose concentration. Values are presented as box plots, in which the box indicates the interquartile range (25th–75th percentile) and the line within the box represents the median. Outliers beyond the typical range are presented as individual points

The primary endpoint of the study, the increase in glucose concentration, calculated as the difference between the maximum glucose level and the baseline for each participant, did not differ significantly between Visits 1 and 2. Mean increases were 75.6 ± 23.6 mg/dL in Visit 1 and 70.0 ± 20.2 mg/dL in Visit 2, with medians and interquartile ranges of 76.0 [60.0–80.0] and 66.0 [56.0–86.0], respectively (*p* = 0.0573, Fig. 3).

### Urinary glucose and sodium excretion In OGTT

A total of 70 and 73 urine samples were obtained during Visit 1 and Visit 2, respectively. Urine samples were categorized according to collection time as follows: −15–0 min before OGTT initiation and 0–30, 30–60, 60–90, and 90–120 min thereafter. Creatinine, sodium, and glucose concentrations were quantified, and creatinine-adjusted sodium and glucose values are presented in Table 2.

**Table 2:** Urinary Glucose and Sodium Ion Concentrations, With Creatinine-Corrected Values

Time (min)	Visit	SampleSize	Cr (mg/dL)	*P* value	Na (mmol/L)	*P* value	Glc (mg/dL)	*P* value	Na / Cr	*P* value	Glc / gCr	*P* value
-15-0	1	*n* = 15	132.2 [78.8-185.2]	0.1934	141.0 [85.0-180.0]	0.6250	6.7 [4.0-7.9]	0.4199	1.0 [0.6–1.7]	0.1309	43.9 [40.4–51.6]	0.7695
	2	*n* = 11	107.5 [64.1-168.3]		111.0 [64.0-192.0]		5.3 [3.4–8.9]		0.9 [0.6–1.8]		46.0 [35.8–56.5]	
**0–30**	**1**	*n* = 3	**91.3** **[44.0-110.4]**	**0.5000**	**101.0** **[101.0-126.0]**	**0.5000**	**2.1** **[1.9–4.6]**	**1.0000**	**1.4** **[0.9–2.3]**	**0.5000**	**50.4** **[43.2–63.4]**	**1.0000**
	**2**	*n* = 9	**65.6** **[21.3-120.7]**		**71.0** **[40.0-89.5]**		**2.8** **[1.2–6.5]**		**1.2** **[0.6–2.3]**		**51.5** **[42.6–62.3]**	
**30–60**	**1**	*n* = 7	**21.6** **[17.6–89.1]**	**0.8750**	**47.0** **[29.0-117.0]**	**0.1250**	**1.4** **[1.1–37.1]**	**0.8750**	**2.1** **[1.2–2.3]**	**0.3750**	**65.0** **[55.9-204.4]**	**0.6250**
	**2**	*n* = 16	**33.1** **[12.0-57.9]**		**53.0** **[24.0-100.3]**		**2.7** **[1.1–6.5]**		**1.8** **[0.9–2.2]**		**71.6** **[53.5–101.0]**	
**60–90**	**1**	*n* = 19	**40.7** **[24.3–56.0]**	**0.3223**	**37.0** **[22.0–99.0]**	**0.1387**	**4.2** **[1.9–8.5]**	**0.3008**	**1.5** **[0.7–1.8]**	**0.1602**	**81.1** **[56.5-277.8]**	**0.4316**
	**2**	*n* = 14	**29.0** **[14.3–92.5]**		**62.0** **[22.8–100.0]**		**2.2** **[1.4–5.4]**		**1.5** **[1.2-2.0]**		**64.7** **[46.8-408.8]**	
**90–120**	**1**	*n* = 17	**82.9** **[18.6–146.0]**	**0.3013**	**77.0** **[42.5–72.0]**	**0.6377**	**6.2** **[2.6–12.0]**	**0.1045**	**1.3** **[0.7–1.9]**	**0.5186**	**68.2** **[47.4-165.2]**	**0.2661**
	**2**	*n* = 19	**18.1** **[12.4–52.6]**		**44.0** **[25.0-103.0]**		**1.6** **[0.9–4.6]**		**1.5** **[0.9–2.8]**		**69.2** **[55.4–96.4]**	
**120-**	**1**	*n* = 9	**35.4** **[15.3–131.0]**	**1.0000**	**41.0** **[26.5–163.0]**	**1.0000**	**2.1** **[0.9–9.1]**	**1.0000**	**1.4** **[1.1–2.3]**	**1.0000**	**57.3** **[51.7–87.2]**	**1.0000**
	**2**	*n* = 4	**23.8** **[23.7–31.0]**		**48.0** **[36.3–70.3]**		**2.9** **[1.1–5.3]**		**2.0** **[1.5–2.3]**		**93.0** **[44.1-207.7]**	
**Ave.**	**1**	*n* = 70	**52.9** **[24.1-130.3]**	**0.0531**	**77.0** **[32.8–135.0]**	**0.1854**	**4.7** **[1.9–8.1]**	**0.0826**	**1.4** **[0.9–1.9]**	**0.1273**	**57.5** **[46.3-125.9]**	**0.4184**
	**2**	*n* = 73	**33.3** **[16.9–90.4]**		**64.0** **[32.5–91.5]**		**2.7** **[1.0-5.7]**		**1.5** **[0.9–2.2]**		**57.7** **[45.2–90.5]**	

Values are shown as median and interquartile range (25th–75th percentiles)

Across all time intervals, no significant differences in creatinine, sodium, or glucose concentrations were observed between visits 1 and 2.

Although the differences were not statistically significant, creatinine-adjusted urinary glucose tended to be higher at Visit 2 than at Visit 1 at all intervals except 60–90 min. In addition, sodium concentrations during − 15–0, 0–30, and 30–60 min showed a nonsignificant trend toward lower values in Visit 2.

### Serum and urinary L-theanine concentrations

**Fig. 4:**
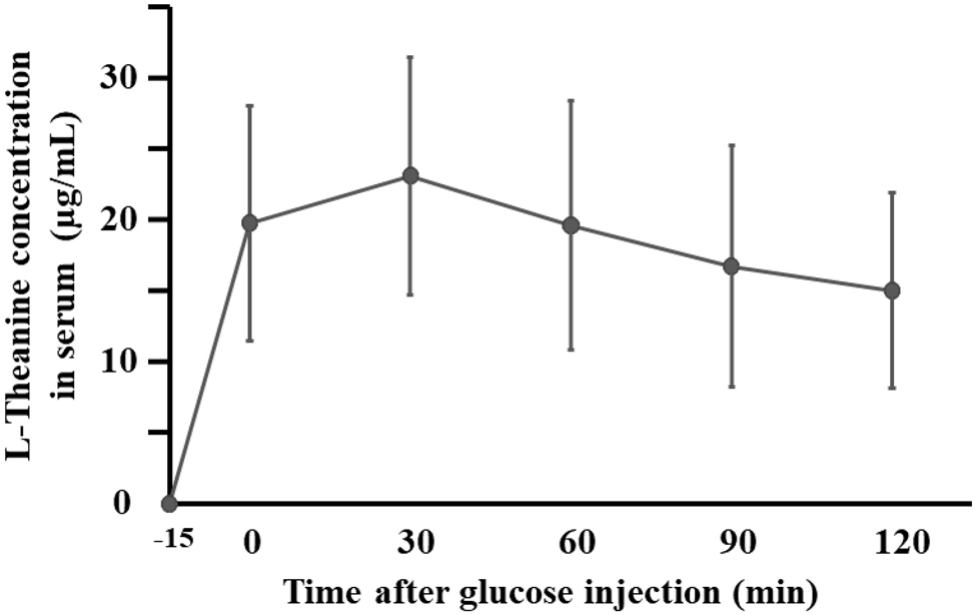
Time-course of Serum L-theanine Concentrations During the OGTT. Because L-theanine was ingested 15 min prior to the start of the OGTT, the time axis begins at − 15 min. Serum L-theanine concentrations are shown as mean ± SD. The sample size was 39

**Fig. 5:**
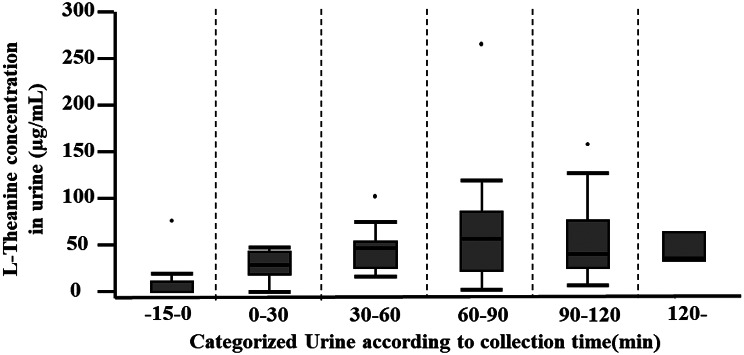
Urinary L-theanine concentrations by collection interval. Urine samples were collected from − 15 to 0 min and subsequently at 30-min intervals. Values are shown as box plots, where the box represents the interquartile range (25th–75th percentile) and the line inside the box indicates the median. Outliers beyond the typical range were plotted individually, and 70 urine samples were collected during visits 1 and 73 during Visit 2 from 39 participants. The number of samples in each collection interval was as follows: visit 1: −15–0 min (*n* = 15), 0–30 min (*n* = 3), 30–60 min (*n* = 7), 60–90 min (*n* = 19), 90–120 min (*n* = 17), and 120 min (*n* = 9). Visit 2: −15–0 min (*n* = 11), 0–30 min (*n* = 9), 30–60 min (*n* = 16), 60–90 min (*n* = 14), 90–120 min (*n* = 19), 120– min (*n* = 4). Statistically significant differences are indicated by asterisks: *p < 0.05, **p < 0.01

Temporal changes in serum and urinary L-theanine concentrations at Visit 2 are shown in Figs. 4 and 5, respectively. Figure 4 presents mean ± SD serum concentrations, and Fig. 5 presents median and interquartile ranges for urinary concentrations.

Serum L-theanine concentration peaked at 30 min, reaching 23.3 ± 8.3 µg/mL, and declined gradually thereafter, with a concentration of 15.0 ± 6.9 µg/mL at 120 min (Fig. 4). L-theanine was detectable in urine even in the −15–0 min interval, with a concentration of 12.5 [0–47.9] µg/mL. The highest median urinary concentration occurred during 60–90 min (60.1 [26.2–88.4] µg/mL), followed by 30–60 min (46.8 [25.4–54.4] µg/mL); the 90–120 min interval showed a similar concentration of 41.1 [25.9–76.4] µg/mL (Fig. 5).

### Changes in serum and urinary amino acids with and without L-theanine

We evaluated differences in the concentrations of amino acids that are substrates of neutral amino acid transporters, such as ASCT2, which may be affected by L-theanine. The concentrations of amino acids other than L-theanine, which could be individually quantified in the serum and urine, are presented in Table 3. In addition to the concentration of each amino acid, urinary amino acid values were obtained after adjusting for creatinine. In serum, significant differences were observed in the concentrations of glutamine (Gln), methionine (Met), alanine (Ala) which were all higher at Visit 2. In the urine, the concentrations of glutamic acid (Glu) increased significantly, and although not statistically significant, all amino acids, except lysine (Lys), Ala, tyrosine (Tyr), proline (Pro), and cysteine (Cys), were excreted at higher concentrations at Visit 2 (Table 3).

**Table 3:** Serum and urinary amino acid concentrations measured during the OGTT

Amino Acids	Visit	Blood (µg/mL)		*P* value	Urea (µg/mL)		*P* value	Urea / Cr		*P* value
Glu	1	9.5	[7.9–11.6]	**0.2637**	8.6	[3.6–16.0]	**0.0934**	0.12	[0.08–0.20]	*0.0304
	2	9.8	[8.3–11.8]		12.3	[7.4–22.2]		0.19	[0.12–0.47]	
Gln	1	59.8	[49.7–67.5]	*0.0275	52.8	[20.5-105.3]	**0.1213**	0.76	[0.60–1.05]	**0.3215**
	2	63.1	[53.8–69.8]		33.4	[15.7–89.4]		0.89	[0.60–1.17]	
Leu	1	14.2	[10.5–17.7]	**0.4905**	6.8	[4.5–11.3]	**0.4217**	0.11	[0.06–0.20]	**0.0582**
	2	13.6	[9.6–17.7]		5.8	[3.4–11.2]		0.12	[0.06–0.27]	
Ser	1	18.9	[13.9–24.2]	**0.4417**	26.0	[12.7–54.4]	**0.7826**	0.46	[0.35–0.65]	**0.2544**
	2	18.2	[14.8–22.4]		21.8	[10.6–48.4]		0.57	[0.36–0.81]	
Met	1	16.7	[6.5–28.8]	*0.0176	21.4	[8.7–37.6]	**0.1823**	0.20	[0.08–0.93]	**0.5530**
	2	20.5	[7.6–33.6]		20.5	[9.6–30.6]		0.28	[0.12–0.78]	
Gly	1	15.9	[12.9–20.0]	**1.0000**	41.4	[18.4–89.9]	**0.4628**	0.83	[0.44–1.27]	**0.0614**
	2	16.8	[14.0-20.3]		35.2	[18.4–88.6]		0.86	[0.48–1.52]	
Val	1	46.6	[25.0-58.5]	**0.5191**	33.5	[8.8–41.7]	**0.9143**	0.26	[0.01–1.24]	**0.1376**
	2	51.7	[20.5–60.6]		30.6	[15.1–41.4]		0.48	[0.11–1.37]	
Lys	1	34.4	[29.4–40.1]	**0.4335**	24.4	[9.8–43.5]	**0.1313**	0.41	[0.24–0.74]	**0.1436**
	2	33.2	[28.7–38.8]		17.5	[8.8–33.0]		0.41	[0.21–0.86]	
Ala	1	33.9	[28.6–39.6]	*0.0399	42.4	[24.4–79.7]	*0.0497	0.98	[0.64–1.19]	**0.5308**
	2	37.2	[32.3–42.1]		34.5	[12.4–77.1]		0.80	[0.49–1.15]	
Thr	1	13.8	[11.4–17.1]	**0.4752**	18.9	[10.3–35.0]	**0.7618**	0.37	[0.23–0.59]	**0.0759**
	2	15.0	[12.2–18.2]		18.9	[10.8–35.2]		0.42	[0.28–0.75]	
Ile	1	9.3	[6.1–11.5]	**0.5739**	8.4	[5.7–11.1]	**0.8600**	0.11	[0.06–0.29]	**0.2000**
	2	9.2	[6.2–12.3]		8.0	[5.7–12.8]		0.17	[0.09–0.35]	
Tyr	1	17.2	[11.3–22.6]	**0.5353**	43.8	[22.7–94.0]	**0.2067**	0.89	[0.64–1.15]	**0.8883**
	2	19.1	[11.3–25.2]		35.5	[15.5–80.2]		0.84	[0.66–1.16]	
Asp	1	5.8	[3.7–8.3]	**0.3282**	23.3	[7.9–51.1]	**0.1661**	0.49	[0.29–0.69]	**0.8831**
	2	5.3	[3.8–7.2]		30.6	[12.7–62.4]		0.51	[0.32–0.85]	
Pro	1	13.9	[11.4–17.0]	**0.1933**	20.8	[9.7–45.2]	**0.9265**	0.43	[0.17-1.00]	**0.3696**
	2	15.3	[12.5–18.3]		16.8	[9.6–34.2]		0.40	[0.20–1.10]	
Cys	1	6.2	[1.6–13.5]	**0.1585**	14.5	[8.3–20.9]	*0.0271	0.20	[0.10–0.45]	**0.9361**
	2	8.5	[2.8–14.6]		11.2	[6.7–15.8]		0.20	[0.11–0.47]	
Phe	1	9.9	[7.5–11.7]	**0.1228**	8.5	[4.6–15.2]	**0.1556**	0.13	[0.09–0.23]	**0.2917**
	2	10.6	[7.9–13.1]		6.8	[3.4–12.5]		0.15	[0.12–0.24]	

The creatinine-corrected values (amino acid/Cr) for urinary amino acids are also presented. Values are shown as median and interquartile range (25th–75th percentiles). A total of 70 urine samples were collected from 39 participants during Visit 1 and 73 during Visit 2. Statistically significant differences are indicated by asterisks: *p < 0.05, **p < 0.01

## Discussion

This study is the first to evaluate the glucose-lowering potential of L-theanine in humans under the limited condition of a single 300-mg oral dose administered to healthy adults prior to an OGTT. At a dose of 300 mg of L-theanine in the present study, no clinically meaningful difference in blood glucose levels may have been observed. In addition, we report, for the first time, the effects of L-theanine ingestion on amino acid profiles in human blood and urine.

To date, the only available information regarding the human pharmacokinetics of L-theanine was reported by Scheid et al. [21]. In their study, healthy adults ingested a 100-mg capsule of L-theanine, resulting in a Tmax of 0.8 h, a Cmax of 24.3 µM (4.2 µg/mL), and a terminal half-life (t1/2) of 1.2 h. In our study, a 300-mg dose produced a Tmax of 45 min, an average Cmax of 23.3 µg/mL, and a t1/2 of 1.8 h. These observations were consistent with those of an earlier report and our previously established pharmacokinetic findings in mice [20]. L-theanine is rapidly absorbed after oral administration in humans, exhibits dose-dependent increases in serum concentration, and is promptly eliminated. Moreover, as described by Scheid et al., L-theanine ingestion increases Glu levels in both serum and urine; our results confirmed the same phenomenon (Table 3). Although ethylamine was not included among the analytes in this study and its elevation was therefore not assessed, based on our results, it is reasonable to assume that the extent of absorption in this study was comparable to that reported by Scheid et al., who demonstrated a bioavailability of approximately > 50%.

Although the present study demonstrated that L-theanine shows no clinically meaningful effect on blood glucose levels, several notable findings emerged from the changes in amino acid composition in the blood and urine. Serum levels of Gln, Met, and Ala increased (Table 3). Previous mouse studies have reported that an elevation in circulating amino acids or administration of specific amino acids can influence glycemia. For example, it has been reported that elevating circulating leucine (Leu) levels can reduce blood glucose levels and influence glucose metabolism [22]. Many of the glucose-lowering effects attributed to such amino acids have been suggested to involve insulin secretion. However, in the present study, no change in Leu concentrations was observed, and there is limited evidence regarding the physiological effects of increased blood concentrations of the amino acids that were elevated. These findings suggest that the glucose-lowering effect of L-theanine identified in our previous mouse study was mediated through a mechanism independent of insulin secretion. Therefore, L-theanine would appear to lower blood glucose levels, at least in part, through mechanisms distinct from those involved in insulin secretion–mediated glucose uptake.

In addition, in the urinary amino acid profile, Glu showed significant increases at Visit 2, whereas aspartic acid (Asp), Thr, Gly, Leu, Ser, and several others exhibited non-significant upward trends (Table 3). These amino acids are substrates of neutral amino acid transporters, such as ASCT2 [23]. It has also been reported that L-theanine is preferentially taken up into cells over Glu through its interaction with Slc38a1, a glutamine transporter [24]. Notably, at 30 min, the time at which serum L-theanine reached its peak, the largest (although not statistically significant) decrease in blood glucose levels was observed (Figs. 2 and 4). Focusing on creatinine-adjusted glucose and sodium concentrations, the decrease in urinary sodium levels was greatest during the 30–60 min interval, when urinary glucose concentrations were highest (Table 2). Although urine volume was not measured, L-theanine was excreted in greater amounts between 30 and 90 min. Taken together, these findings do not contradict our hypothesis that the enhanced activity of neutral amino acid transporters in the proximal tubule facilitates sodium reabsorption, transiently lowers urinary sodium levels, and indirectly modulates SGLT activity.

The effects of L-theanine on blood glucose and glucose metabolism cannot be evaluated solely based on transient urinary glucose excretion, and this study did not assess the impact of continuous administration. Numerous studies have investigated the influence of sustained L-theanine intake on glucose and energy metabolism in mice. Peng et al. reported that administering 100 mg/kg of L-theanine to mice for 10 weeks induced a shift from white adipose tissue to metabolically active brown adipose tissue, thereby improving weight gain and insulin sensitivity [25]. In addition, In the study reported by Kurihara et al. [19], the intervention period was also limited to four weeks, which may have been insufficient; longer-term intake may exert effects on glucose metabolism.

One limitation of the present study is its fixed-sequence, two-period design lacking randomization and placebo/sham control, making it difficult to disentangle the effects of L-theanine from expectancy/placebo effects, time effects, learning effects from the first OGTT, and other period-related confounders. Because the present study did not involve timed or cumulative urine collection, the exact urine volume was not available. Consequently, urinary components were compared on a concentration basis, and accurate excretion amounts could not be evaluated because of potential variation in fluid intake and urine output. Additionally, the study did not examine the long-term dosing effects, and the administered dose may have been insufficient to elicit metabolic changes. Compared with the high doses used in animal studies (100–1000 mg/kg), the human dose used in this study (300 mg; approximately 5–6 mg/kg) was substantially lower. Clinical studies administering 450–900 mg L-theanine correspond to approximately 10–15 mg/kg [11]. Furthermore, evaluations of excessive consumption have used daily doses of 1000 mg, although single-dose amounts remain approximately 250–280 mg [18, 19]. Considering that commercial amino acid supplements often contain more than 1000 mg of a single amino acid per serving, future human studies should use higher doses of L-theanine. From the present results, it was insufficient to adequately assess the effects of increased doses, and these findings suggest that the appropriate dosage of L-theanine as a dietary supplement should be examined in future studies.

## Conclusions

Although L-theanine may have the potential to lower blood glucose levels, no clinically meaningful difference was detected under the conditions and study design employed in the present study. However, further clinical studies are warranted to evaluate the effects of longer-term administration exceeding four weeks or single doses greater than 1,000 mg.

## Data Availability

The dataset(s) supporting the conclusions of this article is(are) included within the article.

## Abbreviations

JSPHCS: Japanese Society of Pharmaceutical Health Care and Sciences
OGTT: Oral glucose tolerance test
EEG: Electroencephalogram
ADHD: Attention deficit hyperactivity disorder
SGLTs: Sodium-glucose linked transporters
AST: Aspartate transaminase
ALT: Alanine transaminase
HbA1c: Hemoglobin A1c
Glu: Glutamic acid
Gln: Glutamine
Met: Methionine
Ala: Alanine
Thr: Threonine
Phe: Phenylalanine
Asp: aspartic acid
Lys: lysine
Ala: alanine
Tyr: tyrosine
Pro: proline
Cys: cysteine
Leu: leucine
